# Supplementary material for: Effect of connection induced upper body movements on embodiment towards a limb controlled by another during virtual co-embodiment
Source: PLoS One. 2023 Jan 5;18(1):e0278022. doi: 10.1371/journal.pone.0278022 (PMC9815592; doi:10.1371/journal.pone.0278022)
Supplement: Attachment — Submitted filename: PLOSONE_Harin_2ndReviewers-m5h2.pdf [file pone.0278022.s003.pdf]

Response to reviewer comments

We have carefully considered all the comments by the reviewer and provided appropriate responses. Please find herewith, our response to reviewer comments.

Please note that the statements in blue are the comments by the reviewer. Our responses are shown in black.

Reviewer 1

Reviewer #1: Thank you for revising the manuscript. The discussion of the revised version is much improved. I still have some concerns regarding the significance and contribution of this paper. My impression is that the authors somehow came out with this joint avatar paradigm and tested it. This paradigm does not seem to be designed to test any hypotheses or answer any unknown research questions.

The key research question motivating this study is the issue of ‘connection’ (and connection related haptic and proprioceptive stimulation). While there is a plethora of studies that combine visual and haptic feedback to test embodiment, in every previous study, the touch is presented visually *on* the artificial limb (being embodied), and correspondingly real haptic and proprioceptive feedback is presented directly *on* the innate limb that the artificial limb replaces. The connection related haptic as well as proprioceptive feedback is never on the limb, but rather on the torso to which the limb is connected. Furthermore, connection related haptic feedbacks are self-induced (by self-generated movements) rather than by external objects (or other limbs) touching the limb under observation. And this is the first study to investigate whether feedbacks (corresponding to the connection) that are not really on the limb, can induce a sense of embodiment towards a moving artificial limb. We have further explained this issue in the introduction.

> *Line: 68-78 (Introduction)*
> *Previous studies by Kalckert & Ehrsson [41][42] have shown that passive synchronous movement of a participant’s finger along with the corresponding finger of a rubber hand (while the participant observes only the rubber hand) induces an equally strong sense of ownership towards the rubber hand compared to the traditional rubber hand illusion which involves visuo-tactile stimulation with a paint brush. However, if we try to induce embodiment towards an artificial limb or a prosthetic limb of an amputee, the passive synchronous movements of the artificial*

limb (or the autonomous prosthetic limb) will not provide proprioceptive feedback of the whole limb movements to the amputee. Limb movements will only lead to forces and hence movements of the body since the artificial limb is physically connected to the user's body. It is unknown whether such connection induced body movements caused by the connection forces can increase the sense of ownership and/or agency towards partner-controlled limbs while the user/amputee observes its movements.

Then, we added the following lines to the introduction explaining the hypotheses. In the discussion section, we added sentences regarding the hypotheses.

Line: 165-174 (Introduction)
"In summary, the purpose of this experiment was to simulate a feeling similar to the movements felt on the torso of an amputee wearing a prosthetic arm when the prosthetic arm moves by itself and to investigate if such movements influence the senses of ownership and agency felt towards the prosthetic arm. Therefore, we made the following two hypotheses regarding the Tied condition in which such upper body movements were present and the Separated condition in which such upper body moves were absent while the partner-controlled arm moved.
H1: Senses of ownership and agency towards the virtual non-controlled arm would be higher in the Tied condition compared to the Separated condition.
H2: Proprioceptive drifts towards the virtual non-controlled arm would be higher in the Tied condition compared to the Separated condition."

Line: (Discussion)
Thus, H1 was supported, but the effects of passive synchronized upper body movements or connection force were not large and limited.

Line: (Discussion)
Thus, H2 was partly supported, but we need further discussion and future investigation.

If the authors wanted to test whether indirect movement and haptic feedback could affect embodiment toward an uncontrolled limb, the pulling force to the partner's shoulder would be unnecessary.

Since our setup provides pulling (as well as pushing) forces to both shoulders of the partner, we are unsure if by "pulling force to the partner's shoulder", you meant the pulling force provided to the shoulder of the controlled arm side or the pulling force provided to the non-controlled arm side of the partner. So, we will answer for both cases below.

<u>If you meant the pulling force to the controlled arm side shoulder of the partner,</u>
We think this is an important limitation of our setup that we missed to explain. You are correct that it is unnecessary. But for our setup to work and provide the overall upper body movement, it was necessary. We added explanation to the limitations of the study section. Thank you for pointing this out.

> *Line: 458-468 (Limitations of the study)*
> *To simulate passive movements on the upper body felt due to a real prosthetic arm being moved, our brace device with support poles provided pushing and pulling forces to the non-controlled shoulder. However, pulling and pushing forces had to be applied to the controlled shoulder as well for this setup to work and provide an overall rotational movement to the upper body since the reaching task involved rotational movements of the upper body to reach targets far away from the avatar. Although these forces to the controlled side makes the simulation deviate from real upper body movements that may be caused by a self-moving/autonomous prosthetic arm, these pulling/pushing forces were necessary for our setup to successfully provide upper body movements to each other in synchrony with the virtual arm controlled by the partner. A more sophisticated force feedback device like a robot exoskeleton setup, may have to be used to simulate a more realistic haptic feedbacks closer to upper body movements caused by the pull of a moving attached autonomous arm and minimize such limitations.*

<u>If you meant pulling force to the non-controlled arm shoulder by the partner,</u>
When a real arm moves, we receive both, a pulling force at the shoulder due to the connection of the limb to our body, as well as the resulting series of indirect movements, which includes the forward movement of the upper torso and a twisting of the upper torso, the resulting twist on the hip, and even forces on the legs. Here we

aim to reproduce/simulate effects of a connection, and hence all the above effects. Our joint body setup was designed keeping this in mind. Therefore, as we want to reproduce a ‘connection’ and related effects as best as possible, we feel the reproduction of the force at the partner’s shoulder is necessary for this.

It is also difficult to imagine when such joint embodiment would occur in the real world. I hope the authors could revise the introduction to explain why the research on ‘joint avatar’ and corresponding ‘joint ownership’ would be important.

We revised the introduction with explanations about why research on ‘joint avatar’ and corresponding ‘joint ownership’/embodiment are important.

> *Line: 153-164 (Introduction)*
> *“Joint/shared/co-embodied avatars and robots can provide means for people with different physical disabilities to combine their strengths with others and work together in one complete avatar/robot to perform complicated tasks effectively while minimizing their physical disabilities at the same time. For example, a person missing a left arm can pair up with a person missing a right arm to co-embody one joint avatar and control different sides together as one whole avatar in VR. However, in such cases, since one half of the body is completely controlled by another, it becomes necessary to figure out ways to increase the sense of embodiment (induce illusory embodiment) to body parts controlled by the partner to minimize discomfort. It is difficult to imagine that two persons are physically connected in the real world. However, the force on the upper body could be conveyed to a person at a distant place with appropriate devices. It is much easier than conveying detailed proprioceptive senses of the whole limb. Thus, our experimental paradigm will contribute to developing joint avatars and robots in the future.”*

If we only think from one participant’s perspective, the person could see a virtual limb conducting reaching movements that were controlled by someone else, and received synchronized pushing forces on the corresponding shoulder. This possibly has some effects on the ratings of ownership and agency. However, the question still is, is this novel, and is this important? I’m not fully convinced.

The novelty is in the fact that we test embodiment in scenarios where, instead of the limb being replaced (by the embodied artificial limb), the feedbacks due to the

connection are felt on the body to which the limb is attached. We hope it is better conveyed now compared to the previously revised manuscript. Importance of joint avatars and this study is also added as mentioned in the answer for the previous comment. Overall, the Introduction section has been mainly revised taking your comments into account. Thank you.

Minor comment:
The authors used he/his to describe the paradigm in the introduction. Although all the participants were male, the paradigm was not designed for male-only. The authors should replace these pronouns.

Our apologies. This issue is now corrected. We changed term “he” to “participant” in the introduction.

Lines: 85, 86
